# Draft Genome Sequence of the Hypervirulent *Klebsiella pneumoniae* Strain hvKP1, Isolated in Buffalo, New York

**DOI:** 10.1128/genomeA.00065-13

**Published:** 2013-03-14

**Authors:** Thomas A. Russo, Steven R. Gill

**Affiliations:** a Department of Medicine, University at Buffalo-State University of New York, Buffalo, New York, USA; b Department of Microbiology and Immunology, University at Buffalo-State University of New York, Buffalo, New York, USA; c Witebsky Center for Microbial Pathogenesis, University at Buffalo-State University of New York, Buffalo, New York, USA; d Veterans Affairs, Western New York Healthcare System, Buffalo, New York, USA; e Department of Microbiology and Immunology, the University of Rochester School of Medicine and Dentistry, Rochester, New York, USA

## Abstract

Hypervirulent variants of *Klebsiella pneumoniae* have been primarily reported in the Asian Pacific Rim, but they are spreading across the globe. We report the sequence of *K. pneumoniae* strain hvKP1, which caused liver-splenic abscesses in an otherwise healthy 24-year-old from Buffalo, NY, which will assist in determining why these variants are more pathogenic than “classic” *K. pneumoniae* strains.

## GENOME ANNOUNCEMENT

A new hypervirulent (hypermucoviscous) variant of *Klebsiella pneumoniae* has emerged. First described in the Asian Pacific Rim in 1986 (1), it is now increasingly recognized in Western countries (2). Its defining clinical features are the ability to cause serious, life-threatening community-acquired infection in younger healthy hosts, including liver abscesses, pneumonia, meningitis, and endophthalmitis, and the ability to metastatically spread from the primary site of infection, which is an unusual feature for enteric Gram-negative bacilli (e.g., extraintestinal pathogenic *Escherichia coli*, “classic” *K. pneumoniae*) in a nonimmunocompromised host (3, 4). A characteristic laboratory feature is that its colonies on an agar plate are hypermucoviscous (which does not necessarily equate to being mucoid), which has been semiquantitatively defined by a positive string test. The string test is positive when a bacteriology inoculation loop or needle is able to generate a viscous string >5 mm in length by stretching bacterial colonies on an agar plate. To date, the majority of hypervirulent *K. pneumoniae* strains have been relatively antimicrobial susceptible. This combination of unique clinical features and a positive string test has been used to distinguish hypervirulent *K. pneumoniae* from “classical” *K. pneumoniae*. In the antibiotic era, most infections due to “classical” *K. pneumoniae*, particularly in developed Western countries, occur in hospitals and long-term-care facilities (5). Recently, these “classic” *K. pneumoniae* strains have received increased notoriety due to their propensity for acquiring antimicrobial resistance determinants (6, 7).

Here, we report the draft genome sequence of the strain hvKP1, a blood isolate from a healthy 24-year-old Vietnamese male from Buffalo, NY, who developed pyogenic liver abscess with metastatic spread to the spleen (8). The genomic DNA of strain hvKP1 was sequenced using an Illumina Genome Analyzer IIx, generating 4,524,424 mapped 65-nucleotide (nt) single-direction reads. The sequence reads were demultiplexed with Casava 1.8, and the genome was assembled into 272 contigs using CLC Genomics Workbench v4.0 (CLC bio). Gene prediction and annotation were carried out using the NCBI Prokaryotic Genome Annotation Pipeline (PGAAP). Annotation and contig sequences are available at GenBank under the accession no. AOIZ00000000 (BioProject PRJNA183508).

Although several genome sequences from “classic” *K. pneumoniae* strains are in the public domain, to date, only one genome sequence from a hypervirulent *K. pneumoniae* strain is publically available (NTUH-K2044, isolated from Taiwan, China) (9). Clearly, there has been a modification to the hypervirulent *K. pneumoniae* phenotype. Yet, an incompletely answered question is what are the mechanisms responsible for this change that have made this variant far more virulent than “classic” *K. pneumoniae* strains, from which it presumably evolved. The availability of additional genome sequences from hypervirulent *K. pneumoniae* strains will facilitate the elucidation of these mechanisms.

### Nucleotide sequence accession number.

Annotation and contig sequences have been deposited in GenBank under the accession no. AOIZ00000000.
